# Real‐World Efficacy of Nab‐Paclitaxel Plus Capecitabine and a PD‐1 Inhibitor in Metastatic or Locally Advanced Esophageal Cancer

**DOI:** 10.1002/cam4.71735

**Published:** 2026-03-20

**Authors:** Yun Wang, Wei‐Jing Zhang, Yun‐Xin Lu, Shi‐Liang Liu, Zhuo‐Yu Zhang, Si‐Min Zhang, Tian‐Wan Wang, Yu Zhong, Dong‐Sheng Zhang

**Affiliations:** ^1^ Department of Medical Oncology Sun Yat‐sen University Cancer Center Guangzhou China; ^2^ State Key Laboratory of Oncology in South China, Collaborative Innovation Center for Cancer Medicine Sun Yat‐sen University Cancer Center Guangzhou China; ^3^ Department of Radiology, Guangdong Provincial Clinical Research Center for Cancer Sun Yat‐sen University Cancer Center Guangzhou China; ^4^ Department of Radiation Oncology, Guangdong Esophageal Cancer Institute Sun Yat‐sen University Cancer Center Guangzhou China; ^5^ Guangdong Provincial Key Laboratory of Food, Nutrition and Health, and Department of Nutrition, School of Public Health Sun Yat‐sen University Guangzhou China; ^6^ Integrated Traditional Chinese and Western Medicine Research Center Sun Yat‐sen University Cancer Center Guangzhou China

**Keywords:** anti‐PD‐1 inhibitor, capecitabine, esophageal cancer, nab‐paclitaxel

## Abstract

**Background:**

Over half of esophageal cancer (EC) cases occur in China, where paclitaxel and platinum agents have become the preferred chemotherapeutic regimen for EC patients. However, there is a clinical need for a non‐platinum‐based therapeutic option.

**Methods:**

Cases were collected from Sun Yat‐sen University Cancer Center between January 2019 and November 2023. Patients with metastatic or locally advanced EC received 4–6 cycles of chemo‐immunotherapy, including nab‐paclitaxel, capecitabine, and a programmed death receptor 1 (PD‐1) inhibitor, with or without subsequent surgery, radiotherapy, or maintenance therapy. The objective response rate (ORR), disease control rate (DCR), progression‐free survival (PFS), pathological complete response (pCR), complete (R0) resection rate, and major pathologic response (MPR) were assessed.

**Results:**

Among the 72 patients retrospectively analyzed, the median PFS was 24.7 months (95% confidence interval: 7.0–42.3 months). 75% of patients were regarded as responders, with an ORR of 66.7% in 42 patients with measurable lesions. The 6‐month, 1‐year, and 2‐year PFS rates (DCRs) were 100%, 96.2%, and 67.9%, respectively. In total, 9 patients underwent surgery, and 29 patients received radiotherapy following the regimen. The R0 resection rate was 77.8%, while both pCR and MPR were 66.7%. The most common adverse events were myelosuppression (27.8%) and liver dysfunction (25.0%).

**Conclusion:**

Our study demonstrated that the combination of nab‐paclitaxel, capecitabine, and a PD‐1 inhibitor was an effective and tolerable strategy for EC patients and a promising first‐line or neoadjuvant treatment option.

## 1 Introduction

According to global cancer statistics in 2022, esophageal cancer (EC) ranks 11th in incidence and 7th in mortality among all cancers worldwide, showing a decline compared to previous years [1, 2]. However, over half of EC cases occur in China, causing over 187 thousand deaths annually [3]. The 5‐year survival rate of EC remains less than 20%, largely due to late diagnosis, which often precludes the opportunity for radical surgery. Therefore, a comprehensive treatment plan that includes chemotherapy, radiotherapy, and surgery is of great necessity for patients with metastatic or locally advanced EC, and chemotherapy may play a pivotal role in this process [4, 5]. On the other hand, neoadjuvant chemotherapy or chemoradiotherapy followed by radical surgery or radiotherapy has become the standard of care for patients with locally advanced EC [6]. Despite these approaches, the prognosis for patients with advanced‐stage EC remains poor, highlighting the urgent need for new therapeutic strategies.

The widespread application of immune checkpoint inhibitors, particularly programmed death receptor 1 (PD‐1) inhibitors, has changed the clinical practice in treating various malignancies including EC [7, 8, 9]. Results from several clinical trials, such as KEYNOTE‐590, CheckMate 649, and ESCORT‐1st, have demonstrated improved prognosis of EC patients treated with anti‐PD‐1 monoclonal antibodies in combination with chemotherapy compared to chemotherapy alone, leading to the integration of PD‐1 inhibitors into the first‐line treatment for advanced EC [10, 11, 12].

Currently, the first‐line chemotherapy options include regimens based on fluorouracil, taxanes, and platinum compounds. In China, preferred regimens for EC patients consist of paclitaxel or its derivative nab‐paclitaxel (also known as albumin‐bound paclitaxel), platinum agents such as cisplatin or nedaplatin, and an anti‐PD‐1 monoclonal antibody. However, the adverse gastrointestinal effects associated with platinum compounds, such as cisplatin, can be severe, and the combination of taxanes and platinum may result in stronger neurotoxicity, challenging the tolerance of some EC patients. Consequently, there is a clinical need for non‐platinum‐based regimens, and the combination of taxanes and fluorouracil has emerged as a new approach for EC patients [13, 14, 15]. Based on the above results, we retrospectively analyzed a total of 72 cases receiving a regimen of nab‐paclitaxel, capecitabine, and a PD‐1 inhibitor as the first‐line treatment for metastatic or locally advanced EC in order to evaluate the real‐world efficacy of this therapeutic strategy.

## 2 Materials and Methods

### 2.1 Study Design and Participants

This is a retrospective real‐world cohort study of EC patients who have received nab‐paclitaxel, capecitabine, and a PD‐1 inhibitor as their first‐line or neoadjuvant treatment at Sun Yat‐sen University Cancer Center between January 2019 and November 2023. The inclusion criteria were: (1) EC confirmed by pathology, including esophageal squamous cell carcinoma (ESCC) and esophageal adenocarcinoma (EAC); (2) metastatic or locally advanced EC; (3) treated with nab‐paclitaxel, capecitabine, and a PD‐1 inhibitor (pembrolizumab, nivolumab, toripalimab, sintilimab, or camrelizumab) at least twice; and (4) at least one re‐evaluation image (usually computed tomography, CT) for efficacy assessment post‐treatment. The standard regimen consisted of 4–6 cycles of chemo‐immunotherapy, after which patients could choose to undergo surgery, radiotherapy, or maintenance therapy (capecitabine, S‐1, apatinib, or a PD‐1 inhibitor) according to their doctors' recommendations. Maintenance therapy was advised for at least 6 months or until disease progression. Tumor response was assessed every 6 weeks through radiographic examinations.

### 2.2 Data Collection

Patients' demographics, diagnosis and previous treatments, blood biochemical data, treatment regimens, pathology and image reports, and adverse events were collected retrospectively from medical records. Efficacy assessment results were categorized as complete response (CR), partial response (PR), stable disease (SD), or progressive disease (PD). Residual tumor was graded according to the Chirieac tumor regression grading system. The objective response rate (ORR), disease control rate (DCR), progression‐free survival (PFS), pathological complete response (pCR), complete (R0) resection rate, and major pathologic response (MPR) were calculated based on these data. Tumor stage was determined according to the eighth American Joint Committee on Cancer staging system, and tumor regression grade (TRG) was identified according to the criteria of the College of American Pathologists/National Comprehensive Cancer Network. AEs were graded according to the Common Terminology Criteria for Adverse Events, version 5.0 [16].

### 2.3 Statistical Analysis

Data were analyzed using IBM SPSS (version 25.0; IBM Corp., Armonk, NY, USA). The chi‐square test was conducted to compare categorical variables. The median PFS and estimated 95% confidence intervals (CIs) were computed by the Kaplan–Meier method, while the log‐rank test was used to compare survival, and the univariable and multivariable logistic regression were used to assess predictive factors for PFS. A two‐sided *p* < 0.05 was regarded as statistically significant.

## 3 Results

### 3.1 Patient Characteristics

This study comprised 72 patients with metastatic or locally advanced EC, of whom 63 (87.5%) were male. As shown in Table 1, the average age was 62 years, ranging from 43 to 79 years. The majority of cases were ESCC (95.8%), although two cases (2.7%) were EAC, and one case (1.4%) was adeno‐squamous carcinoma. In total, 33 cases (45.8%) were metastatic EC (stage IVB, including 5 recurrent cases), while 39 cases (54.2%) were locally advanced EC (stage III–IVA, including 2 recurrent cases). Among the seven recurrent cases (9.7%), six patients had undergone surgery, and one patient had received concurrent chemoradiotherapy (CCRT) prior to this study. Most tumors were poorly (40.3%) or moderately (44.4%) differentiated, and the primary tumor sites were predominantly in the middle and lower thoracic esophagus or the esophagogastric junction.

**TABLE 1:** Patient demographics and clinical data (72 patients).

Variable	No. (%)
Age (years)	62 (43–79)
Gender	
Male	63 (87.5)
Female	9 (12.5)
Primary esophageal cancer	
Cervical and upper thoracic	9 (12.5)
Middle thoracic	34 (47.2)
Lower thoracic or EGJ	26 (36.1)
Multiprimary	3 (4.2)
Histology	
Squamous cell carcinoma	69 (95.8)
Adenocarcinoma	2 (2.8)
Adeno‐squamous carcinoma	1 (1.4)
Differentiation	
Well	3 (4.2)
Moderate	32 (44.4)
Poor	39 (40.3)
Unknown	8 (11.1)
Clinical stage	
III	28 (38.9)
IVA	11 (15.3)
IVB	33 (45.8)
Metastatic site	*n* = 33
Lung	10 (30.3)
Liver	8 (24.2)
Nonregional lymph node	21 (63.6)
Brain	1 (3.0)
Bone	5 (15.2)
Kidney	1 (3.0)
Adrenal gland	1 (3.0)
Peritoneum	1 (3.0)

Abbreviation: EGJ, esophagogastric junction.

### 3.2 Survival Outcomes

**FIGURE 1:**
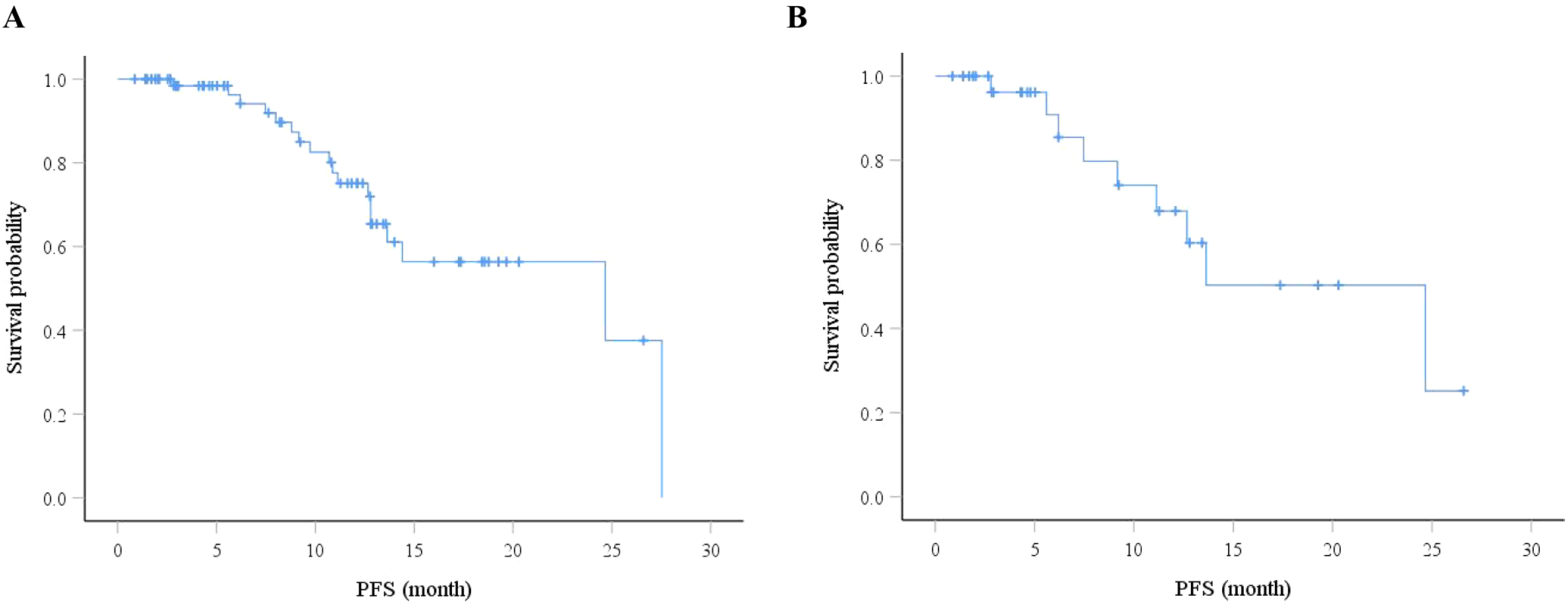
Kaplan–Meier curves for PFS analyses. (A) Kaplan–Meier curve of PFS for all 72 EC patients. (B) Kaplan–Meier curve of PFS for 33 EC patients without subsequent surgery and radiotherapy/CRRT. CCRT, concurrent chemoradiotherapy; EC, esophageal cancer; PFS, progression‐free survival.

The median PFS for all patients was 24.7 months (95% CI: 7.0–42.3 months), as illustrated by the Kaplan–Meier curve in Figure 1A. Among them, 33 patients without subsequent treatment including surgery and radiotherapy/CRRT could also got a same median PFS (24.7 months, 95% CI: 11.9–37.4 months) (Figure 1B). The PFS rates (DCRs) at 6 months, 1 year and 2 years were 100%, 96.2% and 67.9%, respectively (Table 2). No patient deaths have been confirmed, so the overall survival (OS) data is not yet available for analysis.

**TABLE 2:** Efficacy of nab‐paclitaxel plus capecitabine and PD‐1 inhibitor.

Variable	No. (%)
CR	0 (0)
PR	55 (76.4)
SD	17 (23.6)
PD	0 (0)
ORR	55 (76.4)
DCR	
2‐month	72 (100)
4‐month	55 (76.4)
6‐month	45 (62.5)
1‐year	18 (25)

Abbreviations: CR, complete response; DCR, disease control rate; ORR, objective response rate; PD, progressive disease; PR, partial response; SD, stable disease.

No cases of PD were observed at the first efficacy evaluation, but tumor progression was noted in 18 patients (25%) at various later time points, including 2 patients who did not receive maintenance therapy after completing 6 cycles of chemo‐immunotherapy. It should be noted that 42 patients (58.3%) had measurable lesions, and we evaluated efficacy according to the Response Evaluation Criteria in Solid Tumors (RECIST) 1.1 criteria. Among these patients, 66.7% (28/42) achieved a PR with no patients achieving a CR, so the ORR was also 66.7%. For the efficacy evaluation of the remaining 30 patients (41.7%) without target lesions, we calculated the changes in thickness of the primary esophageal tumor and the length of the largest metastatic lesion, following the guidance of specialists from the Departments of Radiologists and Radiation Oncology. We defined patients with a total decrease of over 30% as indicative as ‘responders’ (to the treatment), and 86.7% (26/30) of these patients met this criterion. Taken together, 75% of the 72 patients could be seen as responders.

### 3.3 Subsequent Surgery or Radiotherapy

By the final time point (November 31, 2023), nine patients (12.5%) had undergone surgery following our regimen, including six patients with stage III disease, two with stage IVA disease, and one with stage IVB disease (Table 3). The R0 resection rate was 77.8% (7/9), with the pCR and MPR rates both being 66.7% (6/9). Among the 11 patients with stage IVA disease, the surgery rate was 18.2% (2/11), while 4 patients (36.4%) had not reached the point of requiring further treatment. The remaining five patients (45.5%) received radiotherapy or CCRT, including a 66‐year‐old patient who declined surgery due to concerns about the associated risks. Additionally, one patient with stage IVB disease, characterized by para‐aortic lymph node metastasis, underwent surgery and achieved a local pCR (TRG = 0). Disease progression occurred in two patients (22.2%), including one patient with a TRG of 0 and one with a TRG of 2.

**TABLE 3:** Postoperative and postradiotherapeutic outcomes.

Variable	No. (%)
Patients with subsequent surgery	9/72 (12.5)
Stage III	6 (66.7)
Stage IVA	2 (22.2)
Stage IVB	1 (11.1)
R0 resection rate	7 (77.8)
pCR rate	6 (66.7)
MPR	6 (66.7)
TRG = 0	6 (66.7)
TRG = 1	0 (0)
TRG = 2	2 (22.2)
TRG = 3	1 (11.1)
Disease progression	2 (22.2)
Patients with subsequent radiotherapy/CRRT	29/72 (40.3)
Stage III	11 (37.9)
Stage IVA	6 (20.7)
Stage IVB	12 (41.4)
Disease progression	7 (24.1)
Patients without subsequent surgery or radiotherapy/CRRT	34/72 (76.4)
Stage III	10 (29.4)
Stage IVA	4 (11.8)
Stage IVB	20 (58.8)
Disease progression	9 (26.5)

Abbreviations: CCRT, concurrent chemoradiotherapy; MPR, major pathologic response; pCR, pathologically complete response; TRG, tumor regression grade.

The type of radiotherapy, such as intensity‐modulated radiation therapy, was determined by the radiotherapists according to the extent of the disease and the doses required for radical treatment. A total of 29 patients (40.3%) received radiotherapy or CCRT after our regimen, all of whom continued with chemotherapy or immunotherapy as maintenance therapy. Among these patients, 41.4% (12/29) were at stage IVB. Ultimately, seven patients (24.1%) experienced tumor progression.

**FIGURE 2:**
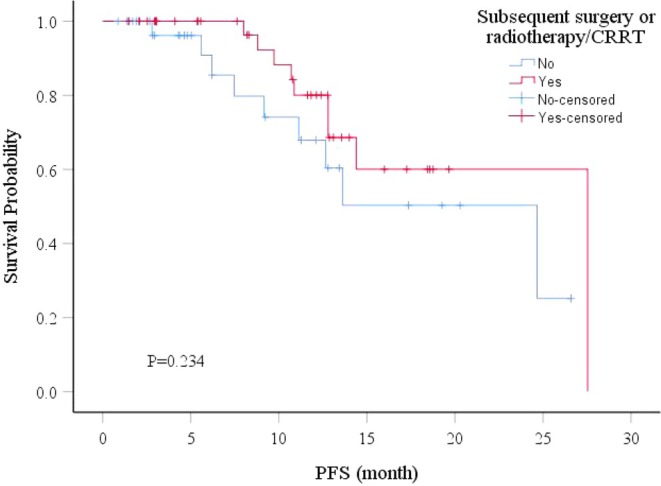
Kaplan–Meier curves of PFS for 72 EC patients with or without subsequent treatment (surgery or radiotherapy/CRRT). CCRT, concurrent chemoradiotherapy; EC, esophageal cancer; PFS, progression‐free survival.

According to the Kaplan–Meier analysis, subsequent surgery and radiotherapy/CCRT showed a trend toward achieving a better prognosis (Figure 2). However, no statistically significant difference was identified.

### 3.4 Prognosis Analysis

To further explore the clinical data potentially associated with patient prognosis, we conducted univariable and multivariable logistic regression analyses based on PFS. The analysis included factors such as patients' gender, age, region, and tumor grade and stage. We also assessed the impact of neutrophil and lymphocyte counts, levels of C‐reactive protein and serum amyloid A in peripheral blood (grouped by the median value), and subsequent treatments (surgery or radiotherapy) on prognosis. As indicated in Table 4, tumor grade emerged as the key factor linked to a poor prognosis for EC patients receiving our treatment regimen.

**TABLE 4:** Effects of factors on PFS in EC patients in the univariate and multivariate cox regression analyses.

Factors	Univariate		Multivariate	
	HR (95% CI)	*p*	HR (95% CI)	*p*
Gender (female vs. male)	0.39 (0.042–3.619)	0.407		
Age (< 60 vs. ≥ 60 years)	0.268 (0.082–0.872)	0.029	0.385 (0.136–1.089)	0.072
Tumor region (cervical and upper thoracic vs. middle/lower thoracic or EGJ/multiprimary)	1.314 (0.233–7.4)	0.757		
Grade (poor vs. well/moderate/unknown)	0.257 (0.078–0.839)	0.024	0.245 (0.084–0.716)	0.01
Stage (III/IVA vs. IVB)	2.944 (0.736–11.783)	0.127		
Neutrophil (low vs. high)	1.027 (0.305–3.463)	0.966		
Lymphocytes (low vs. high)	7.134 (1.16–43.88)	0.034	1.895 (0.671–5.356)	0.228
Neutrophil/lymphocytes (low vs. high)	2.071 (0.331–12.958)	0.437		
CRP (low vs. high)	2.706 (0.628–11.668)	0.182		
SAA (low vs. high)	0.541 (0.138–2.123)	0.378		
Subsequent surgery or radiotherapy (no vs. yes)	0.366 (0.088–1.525)	0.167		

Abbreviations: CRP, C‐reactive protein; EGJ, esophagogastric junction; SAA, serum amyloid A.

### 3.5 Toxicity

Treatment‐related AEs were documented in 69.4% cases (*n* = 50), as detailed in Table 5. No treatment‐related deaths occurred. The most common AEs were myelosuppression (27.8%, *n* = 20, including anemia, leukopenia, and thrombocytopenia) and liver dysfunction (25.0%, *n* = 18, including increased aminotransferase or bilirubin). Furthermore, grade III–IV myelosuppression and liver dysfunction were observed in eight (11.1%) and three (4.2%) cases, respectively. Other AEs occurring in more than 10% of patients included peripheral neuropathy (15.3%, *n* = 11), rash (12.5%, *n* = 9), decreased appetite (11.1%, *n* = 8), and pruritus (11.1%, *n* = 8). Immune‐related AEs, which were considered highly probable, were found in six cases (8.3%) and included rash, pneumonitis, Hashimoto's thyroiditis, myocardial ischemia and adrenocortical insufficiency.

**TABLE 5:** AE related to the treatment.

AEs	Grade I–II No. (%)	Grade III–IV No. (%)
Peripheral neuropathy	10 (13.9)	1 (1.4)
Rash	6 (8.3)	3 (4.2)
Decreased appetite	8 (11.1)	0
Asthenia	7 (9.7)	0
Pruritus	8 (11.1)	0
Hand‐foot syndrome	5 (8.3)	1 (1.4)
Muscular soreness	3 (4.2)	0
Nausea	5 (6.9)	0
Vomiting	6 (8.3)	0
Constipation	3 (4.2)	0
Pneumonitis	3 (4.2)	0
Hashimoto's thyroiditis	1 (1.4)	0
Fever	2 (2.8)	0
Liver injury (Aminotransferase increased)	14 (19.4)	3 (4.2)
Bilirubin increased without increased aminotransferase	1 (1.4)	0
Blood creatinine increased	2 (2.8)	0
Immune‐related myocardial ischemia	1 (1.4)	0
Xerostomia	2 (2.8)	0
Hematochezia	1 (1.4)	0
Epistaxis	1 (1.4)	0
Oral ulcer	1 (1.4)	1 (1.4)
Dizziness	2 (2.8)	0
Myelosuppression	12 (16.7)	8 (11.1)
Abdominal distension	1 (1.4)	0
Aypnia	2 (2.8)	0
Dyspnea	1 (1.4)	0
Hoarseness	1 (1.4)	0
Adrenocortical insufficiency	1 (1.4)	0

## 4 Discussion

EC is a prevalent disease globally, with particularly high incidence and morbidity rates in China. Risk factors for EC include consumption of alcohol, tobacco, hot beverages, and nitrosamines, obesity, and micronutrient deficiencies, highlighting the importance of a healthy lifestyle and dietary habits in preventing this disease [17]. ESCC accounts for nearly 80% of EC cases, whereas EAC comprises less than 20% [5]. Considering the high efficacy of taxanes in treating squamous cell carcinoma, preferred chemotherapy regimens in China typically include taxanes (such as paclitaxel or docetaxel). Nab‐paclitaxel, an albumin‐bound formulation of paclitaxel with a 130‐nm particle size, overcomes several limitations of conventional drugs, including poor solubility and hypersensitivity reactions [18]. As a result, nab‐paclitaxel is increasingly favored as a first‐line chemotherapeutic agent for EC patients in China.

Our medical team retrospectively evaluated the efficacy as well as the safety of a regimen without platinum for patients with metastatic or locally advanced EC in the real world. Because 30 cases receiving this regimen lacked target lesions under the RECIST 1.1 criteria and some previous reports suggested that the decrease of esophageal wall thickness is associated with histological therapeutic effect [19, 20], we assessed efficacy using the modified criterion mentioned above. The efficacy for the remaining 42 cases (ORR = 66.7%) was still evaluated according to the RECIST 1.1 criteria. In summary, we found a high proportion (75%) of responders for this regimen, and its 1‐year DCR was also above 95%. Fortunately, no death was found by the final point of data collection, and the OS analysis was still immature. Taken outcomes of some large phase 3 studies as references, such as ESCORT‐1st (ORR = 72.1%, 1‐year OS = 62.4%) and KEYNOTE‐590 (ORR = 45%, 1‐year OS = 50.5%) [10, 12], our regimen holds favorable therapeutic potential.

For the nine patients who underwent surgery, the R0 resection rate, pCR, and MPR were 77.8%, 66.7%, and 66.7%, respectively. However, we should admitted that the sample size was quite small, so these could be exploratory outcomes. In addition, few severe AEs were observed during the treatment, showing its good safety profile. Taken together, our regimen demonstrated effectiveness and tolerability as a compelling option for first‐line or neoadjuvant treatment in EC patients.

The wide PFS CI (7.0–42.3 months) is probably due to the relatively limited number of cases, the wide range of tumor stages, data heterogeneity (such as different observation times among the patients), and the complex subsequent therapeutic options, including maintenance therapy. In addition, due to the high cost and lack of targeted drugs, most patients refused to undergo genetic testing (molecular diagnosis). These may also be important reasons why our analysis identified tumor grade as the only predictive factor for poor prognosis in the univariable and multivariable logistic regression analyses. Although not statistically significant, we observed a trend indicating that both subsequent surgery and radiotherapy/CCRT provided considerable benefits to patients, underscoring the importance of comprehensive treatment approaches.

It is undeniable that a large number of EC patients suffer from dysphasia, which may limit the applicability of our regimen. However, this treatment offers a relatively mild alternative, avoiding the neurotoxicity and other side effects associated with platinum‐based therapies, particularly for patients in generally poor condition. The most common grade III–IV adverse event was myelosuppression, which was seen in 11.1% of cases. Additionally, the use of oral capecitabine rather than prolonged intravenous administration of 5‐fluorouracil, which often requires a venous catheter, is more convenient and acceptable for Chinese patients.

On the one hand, several reports have demonstrated the strong potential of neoadjuvant immunotherapy plus chemotherapy or chemoradiotherapy for EC patients [21, 22], although further studies are needed before the utility of this therapeutic strategy can achieve a broad consensus. On the other hand, the effectiveness of adjuvant chemotherapy or immunotherapy after surgery or radiotherapy in reducing cancer recurrence remains uncertain, despite some promising results [23, 24]. Given that the majority of the patients in our study chose to receive maintenance therapy following surgery or radiotherapy, we believe that further investigation evaluating OS and PFS in a larger patient cohort would provide a more comprehensive assessment of our regimen, ultimately enhancing our understanding of neoadjuvant and adjuvant immunotherapy.

## 5 Conclusion

Our study revealed that regimens combining nab‐paclitaxel, capecitabine, and an anti‐PD‐1 inhibitor were effective and tolerable for patients with metastatic or locally advanced EC. This clinical practice provided more evidence of a non‐platinum‐based therapeutic option for EC patients as their first‐line or neoadjuvant treatment.

## Author Contributions

Dong‐Sheng Zhang designed the study. Yun Wang, Si‐Min Zhang, Tian‐Wan Wang, and Yu Zhong collected and summarized the data of patients. Wei‐Jing Zhang rechecked the radiographic examination and the tumor stage of each case. Shi‐Liang Liu rechecked the data of radiotherapy of all cases. Yun Wang, Yun‐Xin Lu, and Zhuo‐Yu Zhang analyzed and interpreted the data. Yun Wang drafted the manuscript. Dong‐Sheng Zhang and Yun‐Xin Lu reviewed and revised the paper. All authors have read and approved the submitted version of the article.

## Funding

This work was supported by grants from Tencent Foundation.

## Ethics Statement

This study has been approved by the Ethics Committee of Sun Yat‐sen University Cancer Center. Informed consent was obtained from all patients in this study.

## Consent

The authors have nothing to report.

## Conflicts of Interest

The authors declare no conflicts of interest.

## Data Availability

The data that support the findings of our study are available on request from the corresponding author.
